# Patient representatives’ views on patient information in clinical cancer trials

**DOI:** 10.1186/s12913-016-1272-2

**Published:** 2016-02-01

**Authors:** Pia Dellson, Mef Nilbert, Christina Carlsson

**Affiliations:** 1 Division of Oncology and Pathology, Institute of Clinical Sciences, Lund University, Medicon Village, Scheelevägen 8, S-22381 Lund, Sweden; 2 Clinical Research Centre, Hvidovre Hospital, University of Copenhagen, Kettegård allé 30, 2650 Hvidovre, Copenhagen, Denmark

**Keywords:** Clinical trial, Patient information, Cancer, Patient preferences, Layout, Focus group interview

## Abstract

**Background:**

Patient enrolment into clinical trials is based on oral information and informed consent, which includes an information sheet and a consent certificate. The written information should be complete, but at the same time risks being so complex that it may be questioned if a fully informed consent is possible to provide. We explored patient representatives’ views and perceptions on the written trial information used in clinical cancer trials.

**Methods:**

Written patient information leaflets used in four clinical trials for colorectal cancer were used for the study. The trials included phase I-III trials, randomized and non-randomized trials that evaluated chemotherapy/targeted therapy in the neoadjuvant, adjuvant and palliative settings. Data were collected through focus groups and were analysed using inductive content analysis.

**Results:**

Two major themes emerged: *emotional responses* and *cognitive responses*. Subthemes related to the former included *individual preferences* and *perceptions of effect*, while subthemes related to the latter were *comprehensibility* and *layout*. Based on these observations the patient representatives provided suggestions for improvement, which largely included development of future simplified and more attractive informed consent forms.

**Conclusions:**

The emotional and cognitive responses to written patient information reported by patient representatives provides a basis for revised formats in future trials and add to the body of information that support use of plain language, structured text and illustrations to improve the informed consent process and thereby patient enrolment into clinical trials.

## Background

Enrolment into clinical trials is central to develop, evaluate and implement new cancer treatments. Respect for patient autonomy is central in both health care and medical research, but the regulatory codes are particularly strict in the latter situation. To guarantee autonomy and protect patients from harm, oral and written clinical trial information is provided; thereafter the patient gives an informed consent to participate [1]. Oral information about study concept, aims, randomization, risks and benefits and practical implications is provided by physicians and research nurses. The patient thereafter receives an informed consent form, which consists of two parts, i.e. an information sheet and a consent certificate.

Written patient information is typically developed by study sponsors, who may represent academic researchers or commercial companies, where after the content is assimilated to local/regional standards and to meet the requirements of ethical committees. The World Health Organization (WHO) provides useful and comprehensive informed consent form templates for clinical studies (http://wma.net/en/30publications/10policies/b3/17c.pdf). The written information is typically extensive and should cover e.g. the purpose of the research, the type of intervention, participant selection, voluntary participation, information on trial drug/placebo, procedures and protocol, treatment alternatives, randomization, additional tests/investigations, duration, standards and guidelines, side effects, potential risks and benefits, reimbursements, confidentiality, sharing of results, contact information, and the right to refuse or withdraw from the study at any time without explanation and without compromised medical care. The signature on a consent form is aimed to verify a voluntary decision to participate and to ensure that the patient understands the risks and benefits linked to participation. The consent process traditionally covers all aspects in entirety. The basic principles is to provide a full and comprehensive picture of potential risks and benefits, but complex information risks reducing the informed consent signature to a legal symbol needed to initiate treatment [2–4]. Though patients are generally satisfied with the consent process, the understanding of e.g. study aims, treatment risks and benefits, randomization and research aspects has been demonstrated to be suboptimal [5, 6]. We explored patient representatives’ views and perceptions of written information used in clinical cancer trials through focus groups.

## Methods

### Patient information leaflets

Written information used in four cancer trials for colorectal cancer was used for the study. The studies had completed accrual and evaluated chemotherapy, targeted therapy and radiotherapy or combinations of these, represented phase I-III trials and were performed in different disease settings, i.e. in the adjuvant, the neo-adjuvant and the palliative settings (Table 1). The study information texts varied between 748 and 2075 words and were written in Swedish. The names of drugs and companies were substituted with ‘the drug/treatment regimen’ or ‘the company’ in brackets.

**Table 1:** Features of the clinical trials and the information leaflets

Trial information leaflets	Phase	Treatment intention	Arms/randomization	Words	Headlines	Medical terms explained/unexplained
A	I-II	Neoadjuvant	1/-	2075	20	14/53
B	III	Palliative	2/+	1090	9	3/50
C	I-II	Palliative	1/-	1032	13	0/15
D	III	Adjuvant	2/+	748	1	3/16

### Informants

Patient representatives were recruited from the two Swedish patient associations for gastrointestinal diseases (www.ilco.nu) (www.magotarm.se). Eligibility included prior colorectal cancer, completed treatment for cancer and no current colorectal cancer. The 14 informants included seven males and seven females with a mean age of 67 years, range 51–77 years, who had been diagnosed with colorectal cancer 1–8 years prior to the study. The level of education was primary school in three individuals, limited further education in four and university studies in five individuals with lack of information in two individuals. The informants had experience from different forms of treatment and four of 14 reported participation in a clinical trial related to their cancer diagnosis. The informants received an information letter including the texts from the four selected trials 3–5 days before the focus group interviews, resembling the typical time allowed for decision-making in the clinical setting.

### Focus group interviews and analysis

Three separate focus group interviews were performed; one group with five female participants, one group with five male participants and group with four participants from both sexes. The participants were asked to put themselves in the patients/potential study participants’ perspective. The focus group discussions led by PD (MD, oncologist with previous experience from 4 focus group studies) and CC (PhD, Study Nurse with extensive experience in focus group studies) who alternated as moderator and registrar.

Each group performed four separate interviews of 30–45 min length on each of the four written trial forms. The interviews was initiated by “*I am interested in your opinion and would like you to describe the text from your point of view.*” To open for suggestions for improvements in the texts, we asked follow-up questions, such as: “*What do you understand?* What do you not understand? At the end of the focus group discussions the informants were asked *“How would you prefer to have it phrased?”* and were invited to provide suggestions for improvements in the information leaflets.

The focus group interviews were audio-taped, transcribed verbatim and analysed using inductive content analysis [7]. Herein, the texts were initially read where after meaning units were identified through text searches for words, statements and sentences with relevance for the study aims and perspectives. The distinct meaning units were coded. After this process of de-contextualisation, the coded meaning units were compared and condensed. In parallel, the texts were condensed. Hereafter, two major themes emerged, under each of which two subthemes were defined. The suggested improvements were included in the analysis, but were separately analysed and grouped and did not contribute to the definition of themes and subthemes.

The study was approved by the Ethics Committee of Lund University (346/2007).

## Results

Two major themes were identified, i.e. *emotional responses* and *cognitive responses*. Subthemes related to the former included *individual preferences* and *perceptions of effect*, while subthemes related to the latter major theme were *comprehensibility* and *layout*. The suggestions for improvement provided are separately presented.

### Emotional responses

The informants found the first impression of a text to be important for their motivation to continue reading and to assimilate the information. Individual preferences were frequently referred to and the informants reflected on their different perceptions, which were viewed to differ also in relation to the disease situation, i.e. adjuvant/neoadjuvant versus metastatic, as well as to the type of treatment discussed since the risk-benefit perception is highly variable between individuals. The informants expressed that the content evoked emotions and stress, particularly related to the decision to participate and to potential side effects. A male informant associated signing the patient information to signing a real estate contract. He found this frightening and expressed a need to learn about the potential benefits of the experimental treatment to overcome feelings of reluctance and to raise motivation to learn about the purpose of the trial.

*…I would rather read about side-effects than risks, because you don’t want to take risks (female group, informant 6, trial A)*

*… so that you can check off yourself… now I’ve done this part of the treatment and now I’m going to go into the next phase… How long does this actually take? … This also gives security. (Female group, informant 9, trial D)*

### Cognitive responses

Comprehensibility and layout were repeatedly referred to and were closely related. Compared with other information materials, the trial information was found to be less attractive and failed to raise an interest in the subject. The informants stressed access to information with a focus on layout, which was perceived to be important for comprehension. The informants repeatedly expressed difficulties to grasp the core contents, in particular related to medical terms and complex words. When the informants encountered difficult sections, they either interrupted reading to figure out the meaning, or skipped the section despite uncertainty about the content. The informants asked for information that was structured and easy to access and comprehend, such as including summaries of key contents, e.g. aims, treatment plan and the risk/benefit, and inclusion of graphic elements and use of bullets. Relevant excerpts are presented below.

*I think that the text is far too heavy. If I’d gotten it in my hand I think I would have read one page and then not read any more. I just wouldn’t have managed. (Male group, informant 13, trial A)*

*… (if there are few headings) you have to read through all the text to see if there’s anything that’s important. (Male group, informant 10, trial D)*

*It’s probably always easier to assimilate if you get something like this with colour or some little extra picture or something in the text. (Female group, informant 9, trial D)*

*… it surprises me that when you spend so much time and money and work on a clinical trial, that you don’t consider beforehand how the patients will perceive this… (Male group, informant 12, trial D)*

*You should emphasize texts and set it in different kinds of letters…use bold text and italics about what’s important and what you want to emphasize…You catch the eye right away.(Male group, informant 13, trial C)*

*A picture says an enormous amount. It can be something drawn or a diagram. (Male group, informant 13, trial B)*

### Suggestions for improvement

The informants suggested a total of 44 improvements data not shown. Age, sex and educational level influenced the number of suggestions, with an underrepresentation among the elderly (9 % from informants >75 years), females (36 % from females) and individuals with less education (33 % from individuals without university degrees). Layout suggestions predominated (24 of 44). Suggestions related to an increased appeal (i.e. layout, graphics and accessibility) and better comprehensibility (i.e. structured contents using headings, sections, colours and bullet points and use of plain language). Additional requests related to explanation of medical terms, refined descriptions of treatments and investigations and detailed data on drug characteristics and managements of side effects.

## Discussion

Focus group data based on patient representatives’ views on written clinical trial information identified *emotional responses* and *cognitive responses* as major themes with subthemes related to *emotions* and *side effects* as well as *comprehension* and *layout*. Written patient information should be complete and comprehensive, which at the same times makes it extensive and complex. Templates for informed consent forms are available, but focus on content and completeness rather than presentation format and comprehensibility. Our findings support greater attention to patients’ perspectives, reactions and responses to this information [8]. The identification of emotional responses is supported also by data that suggest that information in plain language increase satisfaction and decrease anxiety and that optimal design needs to take measures of patient satisfaction and health literacy into account [9–11].

Cognitive responses related to comprehension and layout. The layout of the patient information forms studied was representative, but none of the forms contained illustrations or were perceived to have an attractive layout. The informants identified e.g. poor structure, missing headings, lack of illustrations and a use of medical terms that were not explained. The informants underscored the importance of a logical structure for their ability to understand the contents, e.g. related to sequence of different therapies. The length was not identified as an obstacle, but a clear structure, with e.g. sections, headings, bullet points and key messages, was perceived as important. The informants underscored the importance of graphical elements, which represent the preferred mode of information for many individuals.

Consent forms are generally constructed by medical professionals, who may consider correct and complete information more important than layout. However, nine out of ten prefer graphic design and illustrations, which may be particularly relevant for individuals with poor reading skills [12–16]. These different preference have received less attention in medical texts than in e.g. teaching materials and in multi-media presentations [17–19]. Educational research demonstrates that information perceived as attractive increases its potential and readability [10, 20]. Plain language, short sentences, diagrams, pictures and bullet points have also recently in a randomized study been shown to effectively enhance understanding of consent forms irrespective of health literacy level [21]. Collaboration between investigators and patient representatives has been suggested to improve participant comprehension and satisfaction [6]. Hence, development of future simplified and more attractive informed consent forms require collaboration between investigators, regulatory agents, professional writers, illustrators and patient representatives.

Optimally, patient representatives should be involved in the development of information materials prior to the study. Our study used finalized information forms, which had been used to include a considerably number of patients into the trials in question. The colorectal cancer study information sheets used were compared to a guide for written trial information that we developed in collaboration with breast cancer representatives [22]. Only 11–15 of the 52 defined issues in this guide were met in each of the four colorectal cancer information sheets analysed. Themes asked for that were not included in any of the studies included background data for the drugs used in the trials, a handy format and an attractive layout and inclusion of illustrations. Themes that were partly met, but frequently asked for, included descriptions of how side-effects are managed, separate time estimated for the different study procedures and a structured format with clear section and headings in order to facilitate orientation in a long and complex text. This observation is also supported by Kundapura et al. [23] who found that 70 % of the informed consent forms deviated from the regulations defined.

A number of factors, e.g. perceived personal benefit, chance for cure/clinical benefit, practical issues and altruistic perspectives, influence patients’ decisions to participate in clinical trials, but knowledge about how these issues affect decision-making is limited [24, 25]. Personal interaction, discussions and feedback may positively improve readability and influence understanding. In this process decision aids for patients and guidelines for professionals may improve emotional as well as cognitive responses to clinical trial information [26–28].

## Conclusion

Patient representatives identify multiple shortcomings in written information used in clinical cancer trials. The information was perceived as unattractive, incomplete and partly difficult to read with a lack of structure and illustrations. The themes identified-emotional and cognitive responses-may be relevant to consider in the future development of written information materials. Our findings suggest that relatively simple improvements could increase satisfaction and potentially improve the important process of enrolling cancer patients into clinical trials.

### Practice implication

- Emotional and cognitive responses are described among patient representatives when asked to reflect upon written information for clinical cancer trials.
- Simple improvement related to e.g. structure and illustrations are suggested to improve perception and suggest that future information should be developed with the aid from professional writers, illustrators and patient representatives.
